# Genetic variation analysis of PCV1 strains isolated from Guangxi Province of China in 2015

**DOI:** 10.1186/s12917-018-1345-z

**Published:** 2018-02-07

**Authors:** Liang Cao, Wenchao Sun, Huijun Lu, Mingyao Tian, Changzhan Xie, Guanyu Zhao, Jicheng Han, Wei Wang, Min Zheng, Rui Du, Ningyi Jin, Aidong Qian

**Affiliations:** 1 College of Animal Science and Technology, Jilin Agricultural University, Changchun, 130118 People’s Republic of China; 2 Institute of Military Veterinary, Key Laboratory of Jilin Province for Zoonosis Prevention and Control, Academy of Military Sciences, Changchun, 130122 People’s Republic of China; 3 Guangxi Center for Animal Disease Control and Prevention, Nanning, 530001 People’s Republic of China; 4 College of Veterinary Medicine, Jilin University, Changchun, 130062 People’s Republic of China

**Keywords:** Porcine circovirus 1, Genetic variation, Phylogenetic study, Putative recombinant virus

## Abstract

**Background:**

Porcine circovirus type 1 (PCV1) was discovered in 1974 as a contaminant of a porcine kidney (PK-15) cell line and was generally accepted to be nonpathogenic. But recently it was shown to cause lesions in experimentally infected pig fetuses. Serological evidence and genetic studies suggested that PCV1 was widespread in domestic pigs. Thus, the molecular epidemiology and genetic variation of PCV1 are still necessary to understand.

**Results:**

Here 247 tissue samples were collected from piglets in Guangxi Province, China and performed whole-genome sequencing of the PCV1 genome. Thirteen PCV1 strains were sequenced from the samples. Similarity analysis showed that there were 97.8% to 99.6% nucleotide similarity to each other and 97.1% to 99.8% nucleotide similarity to the 40 reference strains. Besides, based on sequence analysis, we found one putative recombinant virus named GXdx84 strain contained the open-reading frame 1 (ORF1) of PCV1 and the ORF2 of PCV2d-2, which was consistent with the results of phylogenetic analysis that compared PCV1 and PCV2 strains. Variation analysis of the amino acids of the capsid protein revealed that the GXyl224 strain, which encoded 235 amino acids, had two amino acids more than other strains. This is the first study to report that a *cap* gene mutation resulted in lengthening of in the gene sequence.

**Conclusions:**

These data contribute to the understanding of PCV1 evolution and molecular epidemiology that will facilitate programs for its control and prevention.

## Background

Porcine circovirus 1 (PCV1) is a small, non-enveloped, circular single-stranded DNA virus with a genome length of 1.7kb. It is a member of the family *Circoviridae* and genus *Circovirus* [1, 2]. PCV has two genotypes: PCV1 and PCV2. PCV1 was first identified as a contaminant in a pig kidney cell culture (PK-15) [3, 4]. PCV2 is the causative agent of porcine circovirus-associated disease (PCVAD) in swine, and result in substantial economic losses for the pig industry in the world [5].

The genome organization of PCV1 and PCV2 are highly similar. The cis-acting and trans-acting replication factors of both viruses are interchangeable for DNA replication [6–8]. The overall DNA sequence similarity within the PCV1 or PCV2 isolations is greater than 90%, while the similarity between PCV1 and PCV2 isolations is 68% to 76%. There are two major open-reading frames, ORF1 and ORF2, which diverge from the Ori expressing four proteins [9]. ORF1 (*rep* gene) is transcribed in a clockwise direction and encodes two viral replication-associated proteins Rep and Rep'. These two proteins are the main factors for initiation of viral DNA replication. ORF2 (*cap* gene) is transcribed in an anti-clockwise direction, and encodes the immunogenic capsid protein which builds the capsid of the virus [10, 11]. In addition to the replication ORF1 and the capsid protein ORF2, another fragment recognized to be involved in modulation of virulence was encoded by ORF3. PCV1 and PCV2 utilize similar initiation and termination signals at comparable locations within the viral genome. However, they differ from each other with respect to specific RNA expression level as well as splice-junction selection is unique to each virus. Previous research shows that thirteen RNAs reported as PCV1, while ten RNAs to be PCV2 during virus replication in PK-15 cells [12, 13]. Besides, the *cap* of PCV2 encodes the viral capsid protein (Cap) and ORF3-RNA which encodes the apoptosis-associated protein. While the functions of the respective protein are the same, ORF3 of PCV1 is 612nt in length, twice the size of PCV2 [13].

Although PCV1 DNA has been isolated from lymph nodes of a piglet with a wasting condition, it is generally accepted that PCV1 is nonpathogenic but widespread in pigs and porcine cell line PK-15 [14, 15]. However, PCV1 can produce pathology in the lungs of porcine fetuses in foetal life [16]. Clinical data shows that PCV1 infection is common in pigs and that pigs can produce antibodies against PCV1 [6, 17, 18].

The main objective of this study is to analyze the prevalence of PCV1 and the evolutionary patterns as well as the relationships among PCV1 genomes isolated from Guangxi Province of China and compare them with data on PCV1 and recombination of PCV1 and PCV2 published worldwide.

## Methods

### Clinical samples

A total of 247 spleen and lymph node samples were collected from piglets (All piglets were euthanized by an anesthetic overdose with the pentobarbital before collected the samples) in Guangxi Province, China, in 2015. All the pigs displayed signs of progressive weight loss, inguinal lymph node edema and hemorrhage, pulmonary edema, and other lesions. Clinical tissues were homogenized for DNA extraction and stored at -80 °C.

### DNA isolation and polymerase chain reaction (PCR)

Viral DNA was extracted using a TIANamp Genomic DNA kit (TIANGEN, Beijing, China). Two pair of specific PCR primers named PCV1-F and PCV1-R, PCV2-F and PCV2-R were designed according to published PCV1 and PCV2 sequences to amplify the complete genome (Table 1). The PCR assays were performed in a 25μL reaction mixture consisting of 3ng tissue-isolated DNA templates, containing final concentrations of 1.25mM MgCl_2_, 2.5μL 10 × PCR buffer, 1mM of each dNTP, 0.5μM of each primer and 2.5U of *Taq* DNA polymerase (TAKARA, Dalian, China). The DNA was amplified with an initial denaturation of 95 °C for 5 min, followed by 35 cycles of amplification (95°C for 30s, 57°C for 30s, and 72°C for 2min) and a final extension of 72°C for 10min.

**Table 1:** Primers used to amplify the whole genomes of PCV

Name	Sequence (5’-3’)	Tm(°C)	Genome position	Size of amplicons	PCV type
PCV1-F	GGTACCCGAAGGCCGATTTG	56.5	920-939	1759bp	PCV 1
PCV1-R	GGGTACCTCCGTGGATTGTTCT	58.5	905-926		PCV 1
PCV2-F	GCCAGAATTCAACCTTAACCTTTCT	63	1430-1454	1769bp	PCV 2
PCV2-R	GAATTCTGGCCCTGCTCCC	61	1421-1439		PCV 2

### Genome cloning and sequencing

The amplified PCR products were separated by electrophoresis on a 1% agarose. The bands were extracted and purified using the AxyPrep DNA Gel Extraction Kit (AxyGene, USA). Then, the PCR products were ligated into the pMD-18T Vector System (Takara Co. Dalian, China), and the recombinant plasmids were sequenced by Takara Co. (Dalian, China).

### Phylogenetic analysis

To understand the genetic relationship between the PCV1 isolates from Guangxi, 40 published genomic sequences were downloaded from GenBank (Table 1). All sequences were aligned with Clustal W [19] and were analyzed. The phylogenetic tree was calculated using the Maximum Likelihood (ML) method with 1000 bootstrap replicates and the genetic distance of *rep* genes, *cap* genes and complete genomes were calculated using the Kimura 2-parameter, Hasegawa-kishino-Yano and Tamura-Nei model respectively by MEGA6 program [20].

### Recombination analysis

Recombination event analysis was carried out by analyzing the complete genome of potential recombinant of GXdx84 since the strain clustered as a separate branch located between PCV1 and PCV2 in the phylogenetic tree based on complete genome as well as *cap* gene and *rep* gene. In the recombination events, possible breakpoint were identified using two programs based on different approaches: the RDP4 (http://web.cbio.uct.ac.za/~darren/rdp.html) [21] and SimPlot(http://sray.med.som.jhmi.edu/RaySoft) [22]. The RDP tests the recombination events by six methods (GENECONV, BootScan, MaxChi, Chimaera, SiScan and RDP) and the setting for each method was adjusted account for the dataset features according to the RDP manual recommendations. Recombination events detected by more than 4 methods, where a significance value<10^-5^ (P-value<10^-5^) and Bonferroni correction were accepted. The recombination signal and sequences of recombination parental lineages were analyzed by SimPlot. SimPlot analysis was performed with three groups of complete genome: a group of major parent (PCV1G, JN398656), a group of minor parent (PCV2b, AF112862) and a group of potent recombinant (GXdx84, KY437725).

### Selection pressure analysis

The selective pressure analysis of genome was assessed by calculating the difference between the dN and dS rates for the aligned *rep* and *cap* genes by using MEGA version 6.0 software [23]. The entropy was used to measure the genetic complexity which was calculated by BioEdit [24]. The difference in entropy was plotted between dN and dS [25]. If the dN rate was higher than the dS rate, i.e., dN–dS>0, the genes would be considered to be under positive selection; otherwise, the genes would be considered to be under purified selection (dN–dS<0). If the dN rate was approximately equal to the dS rate, i.e., dN–dS=0, the genes would be considered to be under neutral evolution [26].

## Results

### Screening for PCV1 prevalence in clinical samples

Clinical samples (n=247) were collected from the lungs, spleen and lymph nodes of apparently healthy pigs (n=172) and sick pigs (n=75) from different regions of Guangxi Province in 2015. Among collected samples, 32 positive samples are PCV1 (positive rate, 12.95%); among these ones, 21 were from the 75 sick pigs (21/75, 28%) and 11 were from the 172 apparently healthy pigs (11/172, 6.39%). Besides of the 247 samples, 214 were PCV2 positive (positive rate 86.6%). 65 samples were from sick pigs (65/75, 86.67%), 149 were from apparently healthy pig (149/172, 86.62%). Both PCV1 and PCV2 were detected in samples from both diseased and apparently healthy pigs.

### Phylogenetic analysis of the PCV1 isolates

**Fig. 1:**
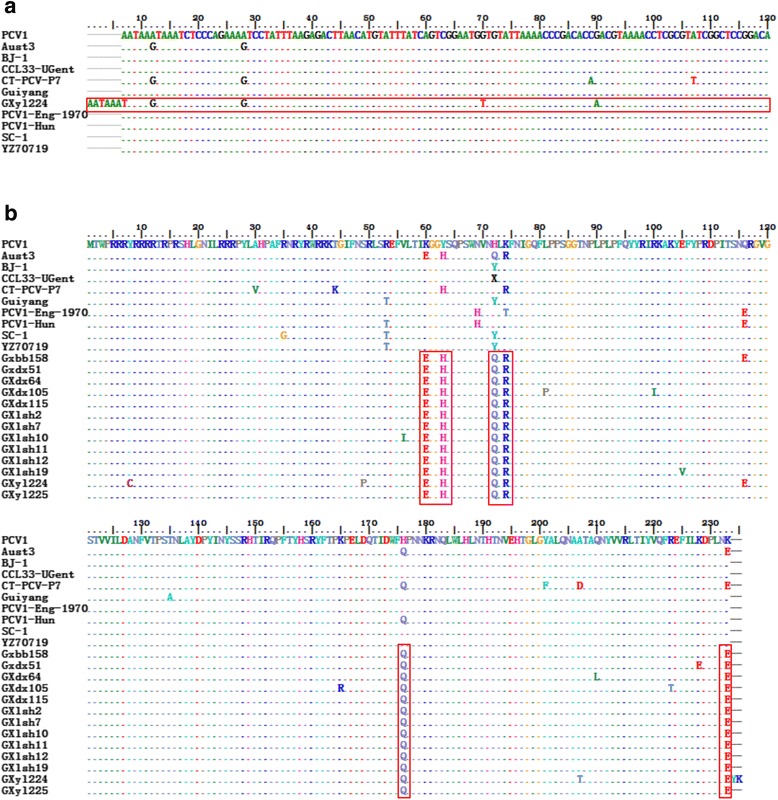
Alignment of the nucleotide sequence and deduced amino acid for the *cap* gene, and Cap protein of partial strains analyzed in this study. Conserver residues are indicated by dashed lines. PCV1 is used as the majority sequence for this alignment (AY193712). **a** The *cap* gene of GXyl224 inside the red box is one wherein the stop codon mutation led to an increase in the number of nucleotides. **b** The major nucleotide mutation sites of Cap protein are presented within the red box

Fourteen complete genome sequences were randomly selected from the 32 positive samples of PCV1 by PCR (Table 2). Of these samples, 13 strains had a whole-genome length of 1759 nucleotides, and only the GXdx84 strain had a genome length of 1757 nucleotides. Regarding the length of the *cap* gene, 12 strains had a length of 702 nucleotides, similar to other strains. But the GXyl224 strains had a length of 708 nucleotides (Fig. 1a). As the stop codon TAA mutated to TAT and added Y and K amino acids were added to the end of the Cap protein. This is the first case of PCV1 wherein a structural gene mutation resulted in gene extension. Considering the complete genome, the similarity among the 13 strains (except GXdx84) in this study ranged from 97.8% to 99.6%. In addition, the nucleotide similarity to the other 40 comparison strains of PCV1 ranged from 97.1% to 99.8%. The percentage of mutations were 13.47% (237 mutations) in the whole genome, 16.24% (114 mutations) in *cap*, and 11.40% (107 mutations) in *rep*. The nucleotide sequence similarity for the complete gene of GXdx84 strains ranged from 86.8% to 89.0% comparing to PCV2 strains used in this study and 86.8% to 89.0% comparing to the other 40 PCV1 strains. The similarity of *rep* gene ranged from 92.2% to 94% in PCV1 and 87.6% to 88.3% in PCV2. While the similarity of *cap* gene ranged from 65.3% to 66.5% in PCV1, and 88.1% to 99.5% in PCV2, especially the similarity to PCV2d-2 was as high as 99.5%. These results indicated that strain of GXdx84 might contain a *rep* came from PCV1 and a *cap* came from PCV2.

**Table 2:** Summary of the complete PCV genomes sequenced used in this study

Strain name	Accesion Number	Country	Collection Date	Genotype	Length(nt)	Source	Linical condition/tissue origin of isolation
BJ-1	FJ475129	Beijing, China	07/5/2009	PCV1	1759bp	GenBank	Lymph nodes, spleen, lung and other tissue materials
PCV1	AY193712	Zhijiang, China	17/2/2003	PCV1	1759bp	GenBank	Lymph nodes, spleen, lung and other tissue materials
PCV1-Hun	KJ408799	Hungary	16/4/2014	PCV1	1759bp	GenBank	lymph nodes, spleen, lung and other tissue materials
PCV1-Eng-1970	KJ408798	United Kingdom	16/4/2014	PCV1	1759bp	GenBank	lymph nodes, spleen, lung and other tissue materials
PCV1-XFD-Beijing	KC447455	Beijing, China	16/4/2013	PCV1	1759bp	GenBank	lymph nodes, spleen, lung and other tissue materials
PCV1/G	JN398656	Harbin, China	31/8/2011	PCV1	1759bp	GenBank	Contaminated PK-15 cell
HZ2006	EF533941	Hangzhou, China	01/5/2007	PCV1	1759bp	GenBank	Lymph nodes, spleen, lung and other tissue materials
NMB	GU799575	USA	28/2/2011	PCV1	1759bp	GenBank	Lymph nodes, spleen, lung and other tissue materials
Jiangsu	GU722334	Jiangsu, China	03/3/2010	PCV1	1759bp	GenBank	Lymph nodes, spleen, lung and other tissue materials
Tian Jin	GU371908	Tianjin, China	06/2/2010	PCV1	1759bp	GenBank	Lymph nodes, spleen, lung and other tissue materials
ZY	KF732857	Guiyang, China	08/1/2014	PCV1	1759bp	GenBank	Lymph nodes, spleen, lung and other tissue materials
DY	KC924758	Guiyang, China	13/8/2013	PCV1	1759bp	GenBank	Lymph nodes, spleen, lung and other tissue materials
GY	KC894933	Guiyang, China	06/8/2013	PCV1	1759bp	GenBank	Lymph nodes, spleen, lung and other tissue materials
Guiyang	KC878437	Guiyang, China	30/7/2013	PCV1	1759bp	GenBank	Lymph nodes, spleen, lung and other tissue materials
ZZ-3	KC733436	Zhengzhou, China	18/6/2013	PCV1	1759bp	GenBank	Lymph node
NJ03	JX566507	Nanjing, China	06/5/2013	PCV1	1759bp	GenBank	Congenital tremors/lymph nodes, spleen, lung and other tissue materials
CT-PCV-P7	AY099501	USA	19/5/2009	PCV1	1759bp	GenBank	Lymph nodes, spleen, lung and other tissue materials
CCL33-UGent	JN133303	Belgium	12/11/2011	PCV1	1759bp	GenBank	Lymph nodes, spleen, lung and other tissue materials
3384	JN133302	United Kingdom	12/11/2011	PCV1	1759bp	GenBank	Lymph nodes, spleen, lung and other tissue materials
HRB-09	GQ449671	Beijing, China	24/10/2011	PCV1	1758bp	GenBank	PK-15 cell culture
PCV3_Rotarix_con1	HM143844	USA	20/6/2010	PCV1	1759bp	GenBank	Lymph nodes, spleen, lung and other tissue materials
SC-10	DQ659154	Guangdong, China	19/6/2006	PCV1	1759bp	GenBank	Pig serum
SC-9	DQ659153	Guangdong, China	19/6/2006	PCV1	1759bp	GenBank	Pig serum
SC-8	DQ494788	Guangdong, China	06/5/2006	PCV1	1759bp	GenBank	Pig serum
SC-7	DQ494787	Guangdong, China	06/5/2006	PCV1	1759bp	GenBank	Pig serum
SC-5	DQ472016	Guangdong, China	24/4/2006	PCV1	1759bp	GenBank	Pig serum
SC-6	DQ472015	Guangdong, China	24/4/2006	PCV1	1759bp	GenBank	Pig serum
SC-4	DQ472014	Guangdong, China	24/4/2006	PCV1	1759bp	GenBank	Pig serum
SC-3	DQ472013	Guangdong, China	24/4/2006	PCV1	1759bp	GenBank	Pig serum
SC-2	DQ472012	Guangdong, China	24/4/2006	PCV1	1759bp	GenBank	Pig serum
NT70719	FJ159693	Jiangsu, China	23/9/2008	PCV1	1759bp	GenBank	Lymph nodes, spleen, lung and other tissue materials
TZ70717	FJ159692	Jiangsu, China	23/9/2008	PCV1	1759bp	GenBank	Lymph nodes, spleen, lung and other tissue materials
SD-73-3	KJ746930	Shandong, China	15/7/2014	PCV1	1759bp	GenBank	Lymph nodes, spleen, lung and other tissue materials
TJ1307	KJ808815	Beijing, China	18/8/2014	PCV1	1759bp	GenBank	Lymph nodes, spleen, lung and other tissue materials
SD-73-1	KJ746929	Shandong, China	15/7/2014	PCV1	1759bp	GenBank	Lymph nodes, spleen, lung and other tissue materials
PK	DQ650650	Jiangsu, China	08/7/2006	PCV1	1759bp	GenBank	Lymph nodes, spleen, lung and other tissue materials
Aust4	AY754015	Australia	21/3/2014	PCV1	1759bp	GenBank	PK-15 cell culture
Aust3	AY754014	Australia	21/3/2014	PCV1	1759bp	GenBank	PK-15 cell culture
SC-1	DQ358813	Guangdong, China	01/3/2006	PCV1	1759bp	GenBank	Pig serum
WB-H-8	DQ648032	Hungary	02/7/2006	PCV1	1759bp	GenBank	Lymph nodes, spleen, lung and other tissue materials
YZ70719	FJ159691	Jiangsu, China	23/9/2008	PCV1	1759bp	GenBank	Lymph nodes, spleen, lung and other tissue materials
YZ70722	FJ159689	Jiangsu, China	23/9/2008	PCV1	1759bp	GenBank	Lymph nodes, spleen, lung and other tissue materials
PCV2a	AF027217	Canada	19/3/2009	PCV2a	1768bp	GenBank	Suspected PMWS/ lymph nodes, spleen, lung and other tissue materials
PCV2b	AF112862	Canada	12/10/2005	PCV2b	1768bp	GenBank	Suspected PMWS/ lymph nodes, spleen, lung and other tissue materials
PCV2c	AF109398	Canada	23/7/2001	PCV2c	1768bp	GenBank	Suspected PMWS/ lymph nodes, spleen, lung and other tissue materials
PCV2d-1(P2425NT)	JX099786	Viet Nam	01/5/2014	PCV2d-1	1767bp	GenBank	Suspected PMWS/ lymph nodes, spleen, lung and other tissue materials
PCV2d-2(CS5)	KX161667	Hunan, China	05/6/2016	PCV2d-2	1767bp	GenBank	Suspected PMWS/ lymph nodes, spleen, lung and other tissue materials
GXdx115	KX827778	Guangxi, China	00/7/2015	PCV1	1759bp	This study	Lungs, spleen, and lymph nodes
GXdx105	KX827779	Guangxi, China	00/7/2015	PCV1	1759bp	This study	Lungs, spleen, and lymph nodes
GXdx64	KX827780	Guangxi, China	00/7/2015	PCV1	1759bp	This study	Lungs, spleen, and lymph nodes
GXdx51	KX827781	Guangxi, China	00/7/2015	PCV1	1759bp	This study	Lungs, spleen, and lymph nodes
GXbb158	KX827782	Guangxi, China	00/7/2015	PCV1	1759bp	This study	Lungs, spleen, and lymph nodes
GXyl225	KX827783	Guangxi, China	00/7/2015	PCV1	1759bp	This study	Lungs, spleen, and lymph nodes
GXyl224	KX827784	Guangxi, China	00/7/2015	PCV1	1759bp	This study	Lungs, spleen, and lymph nodes
GXlsh19	KX827785	Guangxi, China	00/7/2015	PCV1	1759bp	This study	Lungs, spleen, and lymph nodes
GXlsh12	KX827786	Guangxi, China	00/7/2015	PCV1	1759bp	This study	Lungs, spleen, and lymph nodes
GXlsh11	KX827787	Guangxi, China	00/7/2015	PCV1	1759bp	This study	Lungs, spleen, and lymph nodes
GXlsh10	KX827788	Guangxi, China	00/7/2015	PCV1	1759bp	This study	Lungs, spleen, and lymph nodes
GXlsh7	KX827789	Guangxi, China	00/7/2015	PCV1	1759bp	This study	Lungs, spleen, and lymph nodes
GXlsh2	KX827790	Guangxi, China	00/7/2015	PCV1	1759bp	This study	Lungs, spleen, and lymph nodes
GXdx84	KY437725	Guangxi, China	00/7/2015	PCV1/2d	1757bp	This study	Lungs, spleen, and lymph nodes

We also identified several novel amino acid substitutions in the ORF2 genes from PCV1 isolated in this study. The PCV1 isolates showed 6 amino acid substitutions differed from the PCV1 reference strains: 60 (K to E), 63 (Y to H), 72 (H to Q), 74 (K to R), 176 (H to Q) and 233 (K to E) (Fig. 1b).

**Fig. 2:**
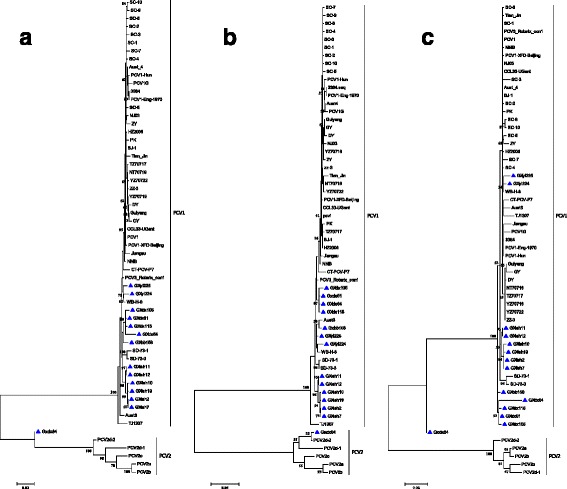
Phylogenetic tree analysis based on the nucleotide sequences of the complete genome (**a**), *cap* gene (**b**), and *rep* gene (**c**), performed using the ML method. Numbers along the branches indicate the percentage of confidence in the ML analysis. Only bootstrap support values of >50% are indicated. PCV1 strains isolated in this study are denoted by triangle (▲)

To understand the genetic relationship among the PCV1 isolates in this study, a maximum likelihood (ML) phylogenetic tree was constructed with the 14 strains collected from Guangxi Province, 40 other comparison PCV1 strains, and 4 PCV2 strains with complete genomic nucleotide sequences available in GenBank. Except for the GXdx84 strain, all isolated strains belonged to the PCV1 and showed geographical differences, but these differences were not evident. In particular, the ML phylogenetic tree analysis showed that the GXdx84 strain was different from the other strains (Fig. 2). The GXdx84 strain was located in the branch of neither PCV1 nor PCV2 strains as shown by the whole nucleotide analysis. With regard to the *cap* and *rep* gene of the samples, *cap* of the strain was located in the branch of PCV2, and *rep* of the strain was located in the branch of PCV1 but was distantly related to other strains of PCV1. These results indicated that the GXdx84 strain might have underwent recombination.

### Recombination analysis

**Fig. 3:**
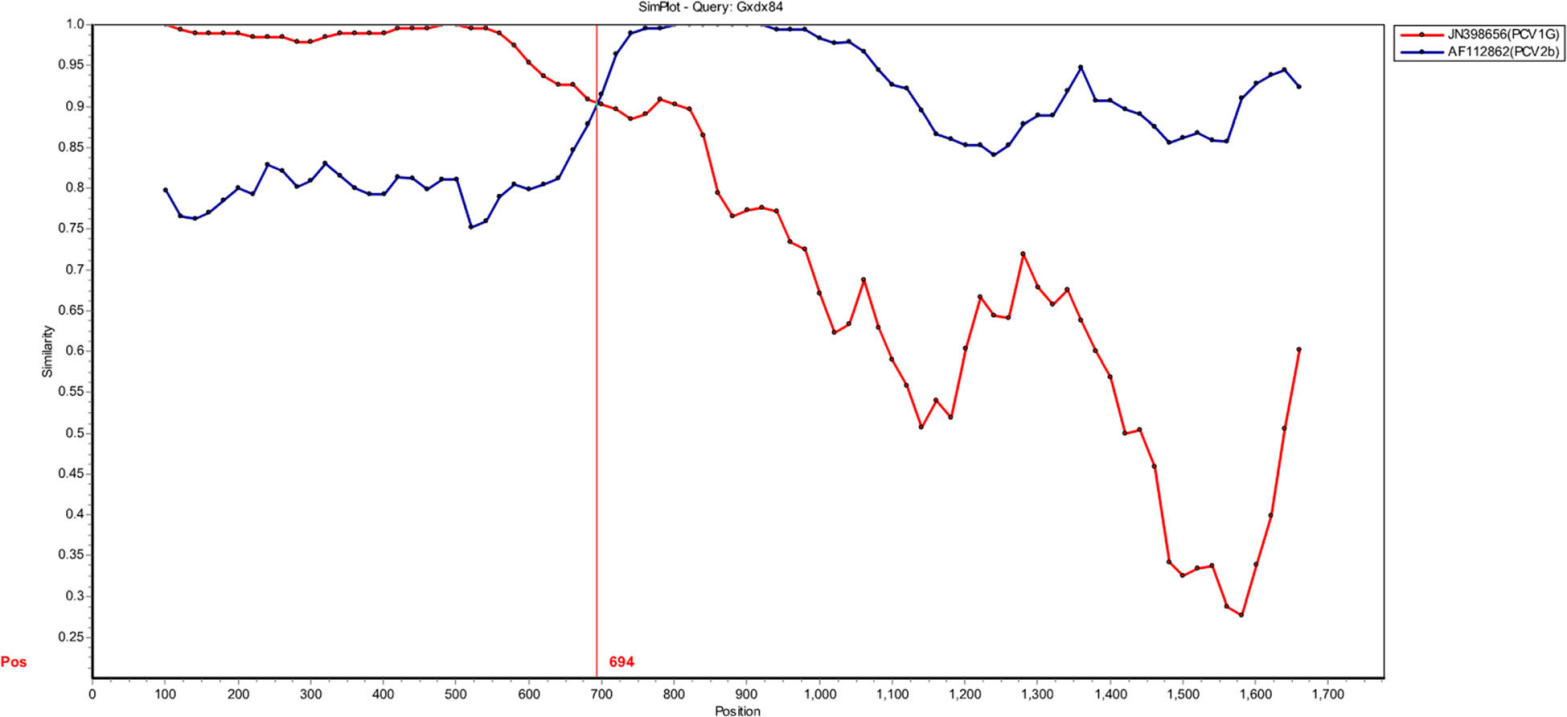
Simplot analysis the recombination events. One recombination event might be occurred in GXdx84 strain with the PCV1G strain (JN398656) and PCV2b strain (AF112862) as two parent groups. The Y–axis refers to the percentage of similarity. The X–axis refers to the nucleotide position in alignment. The crossed point might be the potential location of recombination event

The putative recombination events were identified using the Recombination Detection Program (RDP). For 14 PCV1 isolation strains, one recombination event was detected, in which the GXdx84 strain (P-value=1.54×10^-13^) with the potential parental of PCV1 subtype strain PCV1G and PCV2 subtype strain PCV2b. The number and location of the breakpoints were also determined using similarity plots. When the PCV1G and PCV2b strains were used as potential parental strains, they shared one breakpoint location at 694nt (Fig. 3). It indicated that PCV2b and PCV1G strains might be the minor and major parents of GXdx84 strain.

### Selection-pressure analysis of PCV1

**Fig. 4:**
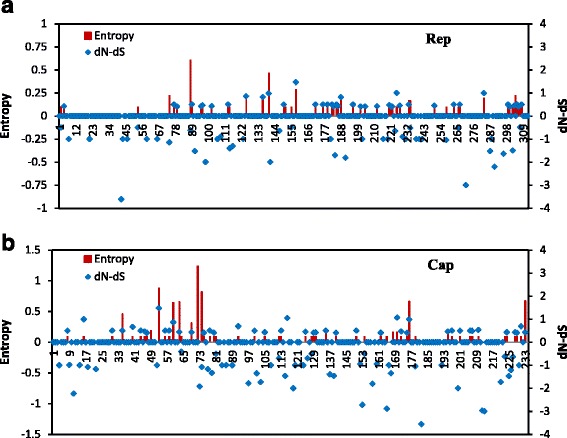
Plot for the difference between non-synonymous and synonymous rates (dN–dS) and amino acid entropy rate for the *rep* (**a**) and *cap* (**b**) genes

The selection-pressure of PCV1 strains was analyzed by calculating the difference in non-synonymous substitution (dN) and synonymous substitution (dS) rates for *rep* and *cap*. The differences of dN–dS were -0.09127±0.0251 for the *rep* gene and -0.2111±0.0541 for the *cap* gene. These results suggested that the *rep* gene and the *cap* gene of PCV1 are under purified selection. Moreover, entropy was coupled with dN–dS to indicate diversity and complexity. The vast majority of Rep and Cap protein amino acid residues exhibited low level or zero entropy (Fig. 4). It indicated that the Rep and Cap protein had low complexity. Only the Cap protein amino acid sequences which were at 50-80, 161-180 and 230-233 had high complexity.

## Discussion

PCV1 is considered to be non-pathogenic and economically unimportant and therefore, little is known about its epidemiology and worldwide distribution. More research on PCV1 has focused on the chemic vaccine of PCV1-2 to prevent PCV2 infected in pigs [27, 28]. Recent research found that PCV1 can replicate efficiently and produce pathology in the lungs of porcine fetuses and have a certain impact on porcine alveolar macrophages [16]. It is difficult to rule out the potential damage of PCV1 to the immune system of piglets.

Recombinant events of PCV among different genotypes or different virus isolates have been reported, including recombinant event between PCV2a and PCV2b, and between PCV1 and PCV2a. In this study, we found a recombination GXdx84 strain which was recombinant by PCV1 and PCV2d-2 had a breakpoint at 694nt. Previous research found that the *rep* gene of the PCV2 can be divided into three regions, and the third region has the highest level of selective pressure which makes the fragment more changeable than the other two [1, 29]. The PCV1 and PCV2 are highly similarity of *rep* gene and this might be the reason why recombinant event occurred at 694nt.

Most studies on the genetic characterization of PCV2 were based on the *cap* gene, which is the ideal marker for phylogenetic analysis because this region is considered to be the most variable region in the PCV2 genome and the same phylogenetic tree constructed on the basis of the full genome can be reconstructed with ORF2. However, analysis based on the complete genome is necessary, as it may help identify genetic variability, particularly in terms of recombination events. In this study, genotypic analysis based on the *cap* gene and complete genome resulted in different phylogenetic trees. The GXdx84 strain was divided into different branches of the phylogenetic tree. Analysis of the *cap* and *rep* genes showed that the new strain was clustered in *cap* in PCV2 and in *rep* in PCV1. Besides, *cap* of the GXdx84 strain was highly similar to the genotype of PCV2d-2. In a recent report from China, the positive rate of PCV2d out of PCV2 positive samples ranged from 55% (22/40) [30] to 68.2% (45/66) [31]. It had become the predominant PCV2 subtype [32]. PCV2d can be classified into PCV2d-1 and PCV2d-2, with substantial genetic divergence between the two subtypes. PCV2d-1 strains were first identified in China in 2002 [33], whereas PCV2d-2 strains were first identified in China in 2008 and had been linked with increased virulence [34, 35].

As for the isolated PCV1 strains, the *cap* gene in the GXyl224 strain spanned 708 nucleotides (codes 235aa), while the other PCV1 strains spanned 702 nucleotides (codes 233aa). This was the first case to report that the capsid protein contains 235 amino acids. Previous studies suggested that the immunoreactive regions of the capsid protein were potential candidate regions involved in the emergence of PCV2 variants [36, 37]. Although PCV1 has no obvious pathogenicity, clinical trials have shown that PCV1 and PCV2 mixed infection generally exhibits the presence of a recombinant strain [38]. Unfortunately, we have not isolated the virus. In the future, we will aim to separate the virus and study its pathogenicity.

In the present study, we also analyzed the selective pressure by calculating the dN and dS rates and the entropy. Very low levels of variability were detected at the nucleotide and amino acid level. Further, the dN–dS values showed that most codons are under neutral or negative selection [39, 40]. Another previous study showed that the average genomic substitution rate for PCV1 was two-fold lower than that for PCV2 [41], which indicated that the PCV1 genome lacked variation. Entropy of Cap and Rep protein coupled with a low complexity, but the Cap protein amino acid sequence which were at 60-80, 161-180 and 230-233 had high complexity (Fig. 4). It was reported that Cap protein was the main antigenic determinant of the PCV2, and contained different epitopes within residues 47-85, 165-200 and the last of the four C-terminal amino acid of the PCV2 capsid protein [42]. Three high complexity regions of PCV1 researched in this study might be the epitopes regions of PCV1. Besides, the Cap protein of GXyl224 coded 235 amino acids, which is two amino acids more than other strains. By analysising the antigenic of GXyl224 by IEDB (http://tools.iedb.org/bcell/), we found that the last six amino acids (230-235aa) were the potential epitope region, other strains of PCV1 were the last four amino acid (230-233aa). Two additional amino acids may result in increased antigenicity of Cap protein, which still need further verification.

## Conclusion

In summary, we have sequenced 14 strains suspected to be PCV1 from Guangxi Province, China. ML phylogenetic tree analysis showed that 13 of these strains belonged to PCV1 and 1 strain named GXdx84 belonged neither to PCV1 nor PCV2: it was a chimeric PCV1 and PCV2 strain. In addition, another stain named GXyl224 with ORF2 spans 708 nucleotides by gene sequencing. This is the first study to report that a *cap* gene mutation resulted in lengthening of in the gene sequence. The correlation between epitope mutations and pathogenicity as well as immunogenicity of GXyl224 and GXdx84 needs further investigation.

## Abbreviations

dN: Non-synonymous substitution
dS: Synonymous substitution
ORF: Open-reading frame
PCV: Porcine circovirus
PMWS: Post-weaning multi-systemic wasting syndrome
RDP: Recombination detection program
